# Supplementary material for: Sequential sequestrations increase the incorporation and retention of multiple growth factors in mineralized collagen scaffolds
Source: RSC Adv. Author manuscript; Available in PMC 2021 Mar 24. (PMC7990239; doi:10.1039/d0ra03872e)
Supplement: Supplemental Information [file NIHMS1632602-supplement-Supplemental_Information.pdf]

Electronic Supplementary Material (ESI) for RSC Advances.
This journal is © The Royal Society of Chemistry 2020

Supporting Information

Sequential sequestrations increase the incorporation and retention of multiple growth factors in mineralized collagen scaffolds

Aleczandria S. Tiffany, Marley J. Dewey, Brendan A.C. Harley

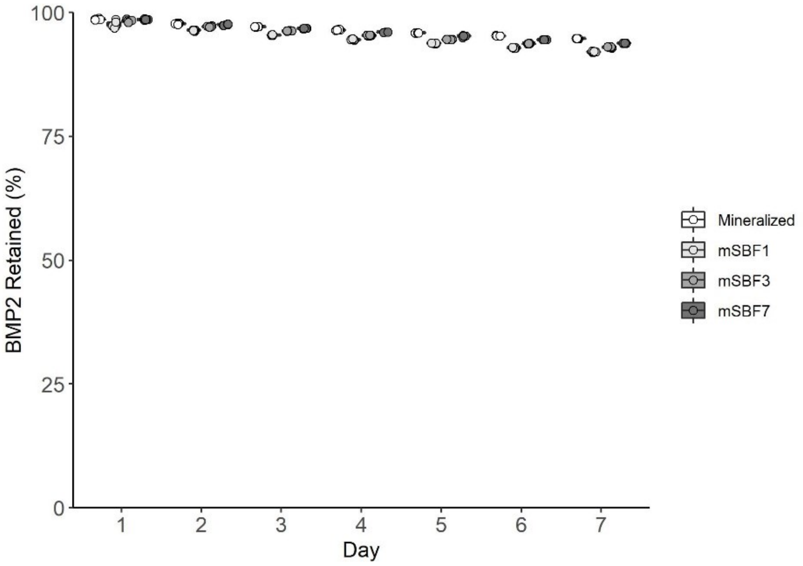

Supplemental Figure 1: Full BMP2 retention plot for data presented in Figure 1C. Boxplots overlaid with individual data points are used to represent data. Retention of BMP2 in scaffolds with the y-axis starting at 0%.

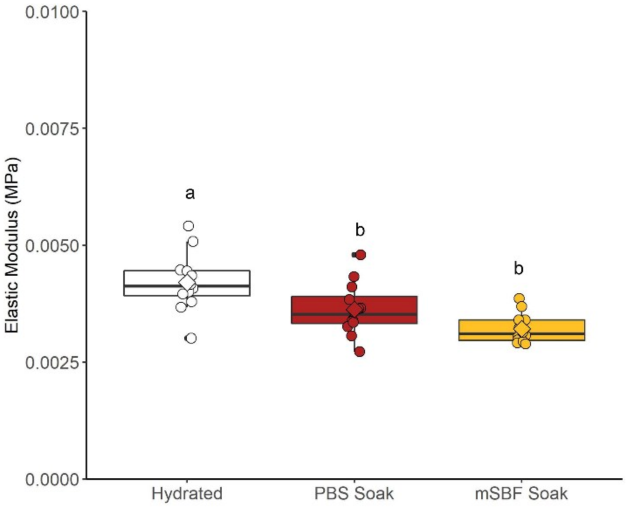

Supplemental Figure 2: Compression testing of hydrated, mSBF soaked, and PBS soaked scaffolds. Boxplots overlaid with individual data points are used to represent data. Elastic modulus of treated scaffolds. All scaffolds groups are softer than 5 kPa. Groups that share a letter are not significantly different ($p<0.05$).

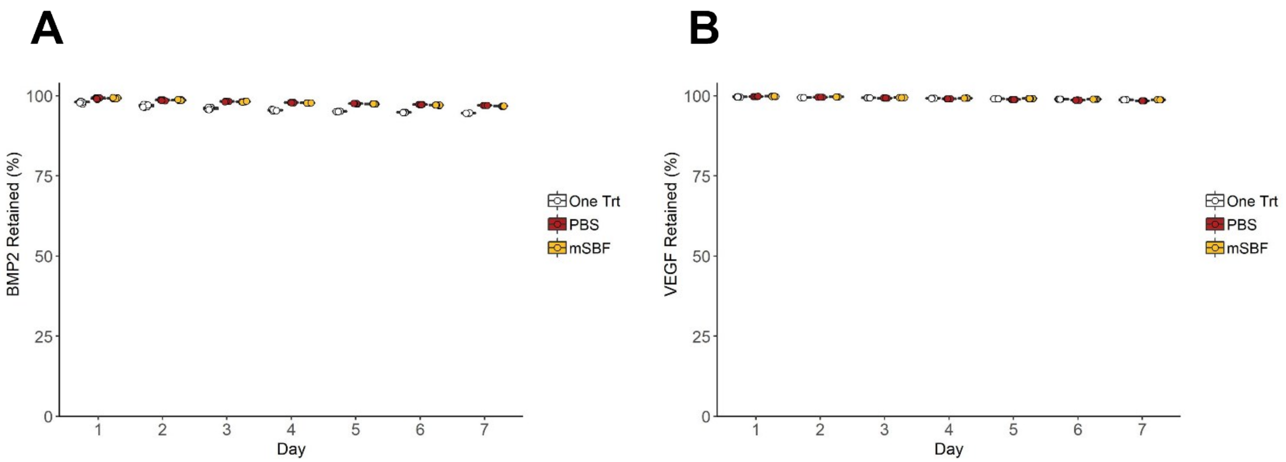

Supplemental Figure 3: Full retention plots for data presented in Figure 2. Boxplots overlaid with individual data points are used to represent data. (A) Retention of BMP2 in scaffolds with the y-axis starting at 0%; corresponding to data presented in Figure 2C. (B) Retention of VEGF in scaffolds with the y-axis starting at 0%; corresponding to data presented in Figure 2E.

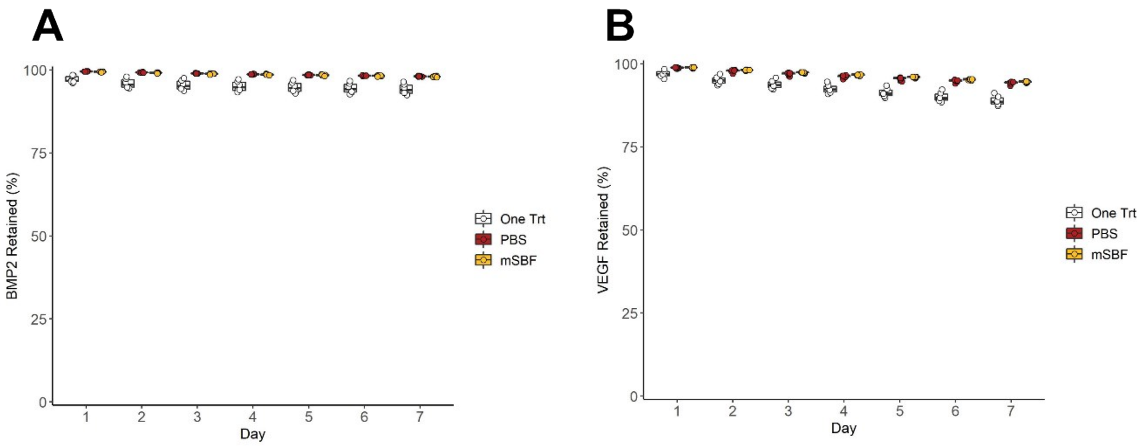

Supplemental Figure 4: Full retention plots for data presented in Figure 3. Boxplots overlaid with individual data points are used to represent data. (A) Retention of BMP2 in scaffolds with the y-axis starting at 0%; corresponding to data presented in Figure 3C. (B) Retention of VEGF in scaffolds with the y-axis starting at 0%; corresponding to data presented in Figure 3E.

Supplemental Table 1. PCR primers and assay IDs.

Transcript	Supplier	Assay ID
18S	ThermoFisher (Taqman)	Hs99999901_s1
PPIA	ThermoFisher (Taqman)	Hs04194521_s1
PDGF	ThermoFisher (Taqman)	Hs00966522_m1
HIF1a	ThermoFisher (Taqman)	Hs00153153_m1
ANG1	ThermoFisher (Taqman)	Hs00919202_m1
ANG2	ThermoFisher (Taqman)	Hs00169867_m1

Supplemental Table 2. Summary statistics for PDGF and HIF1a PCR data

		Platelet Derived Growth Factor		Hypoxia Inducible Factor 1α	
**Day**	**Group**	**Fold Change***	**Sample Size**	**Fold Change***	**Sample Size**
1	Blank	1.310 ± 2.086	7	3.565 ± 4.796	7
1	mSBF	1.246 ± 0.902	8	1.902 ± 0.696	8
1	One Trt	2.191 ± 0.921	7	2.208 ± 0.845	8
1	Soluble	0.440 ± 0.199	8	1.363 ± 0.417	8
4	Blank	2.461 ±1.381	7	3.645 ± 3.468	8
4	mSBF	3.592 ± 2.005	7	1.248 ± 0.379	8
4	One Trt	18.95 ± 28.48	4	10.76 ± 9.932	8
4	Soluble	4.430 ± 3.353	8	2.226 ± 1.506	8
7	Blank	2.123 ± 2.003	8	3.014 ± 5.937	8
7	mSBF	1.624 ± 0.654	6	0.647 ± 0.126	8
7	One Trt	3.619 ± 1.184	5	1.916 ± 1.561	8
7	Soluble	2.514 ± 1.543	6	1.249 ± 1.669	8

*mean ± standard deviation.

Supplemental Table 3. Summary statistics for ANG1 and ANG2 PCR data

		Angiopoietin 1		Angiopoietin 2	
**Day**	**Group**	**Fold Change***	**Sample Size**	**Fold Change***	**Sample Size**
1	Blank	5.360 ± 5.702	7	1.104 ± 1.462	7
1	mSBF	4.176 ± 1.991	8	0.737± 0.290	8
1	One Trt	2.182 ± 1.427	4	0.455 ± 0.186	8
1	Soluble	5.309 ± 4.315	8	0.302 ± 0.086	8
4	Blank	2.508 ± 2.410	7	2.742 ± 2.994	8
4	mSBF	2.030 ± 0.550	7	3.865 ± 7.248	8
4	One Trt	4.324 ± 3.557	3	15.12 ± 12.86	8
4	Soluble	2.126 ± 1.365	7	1.251 ± 0.656	8
7	Blank	1.035 ± 0.335	6	6.024 ± 12.18	8
7	mSBF	1.495 ± 0.447	8	0.936 ± 0.286	8
7	One Trt	2.335 ± 1.438	4	4.058 ± 6.275	8
7	Soluble	1.718 ± 1.195	6	1.666 ± 1.115	7

*mean ± standard deviation.
